# Ultrathin Covalent Organic Framework Nanosheets/Ti_3_C_2_T_x_-Based Photoelectrochemical Biosensor for Efficient Detection of Prostate-Specific Antigen

**DOI:** 10.3390/molecules27196732

**Published:** 2022-10-09

**Authors:** Nanjun Li, Chongyang Wang, Liangjun Chen, Cui Ye, Yongwu Peng

**Affiliations:** College of Materials Science and Engineering, Zhejiang University of Technology, Hangzhou 310014, China

**Keywords:** covalent organic framework nanosheets, Ti_3_C_2_T_x_, composites, photoelectrochemical biosensor, prostate-specific antigen

## Abstract

Designable and ultrathin covalent organic framework nanosheets (CONs) with good photoelectric activity are promising candidates for the construction of photoelectrochemical (PEC) biosensors for the detection of low-abundance biological substrates. However, achieving highly sensitive PEC properties by using emerging covalent organic framework nanosheets (CONs) remains a great challenge due to the polymeric nature and poor photoelectric activity of CONs. Herein, we report for the first time the preparation of novel composites and their PEC sensing properties by electrostatic self-assembly of ultrathin CONs (called TTPA-CONs) with Ti_3_C_2_T_x_. The prepared TTPA-CONs/Ti_3_C_2_T_x_ composites can be used as photocathodes for PEC detection of prostate-specific antigen (PSA) with high sensitivity, low detection limit, and good stability. This work not only expands the application of CONs but also opens new avenues for the development of efficient PEC sensing platforms.

## 1. Introduction

The detection of tumor markers is important for early clinical diagnosis of cancer [1,2,3]. Prostate cancer is one of the most common and fatal diseases in humans. Prostate-specific antigen (PSA) is one of the most reliable cancer markers for early diagnosis of prostate cancer. PSA levels in normal human body serum are below 4 ng mL^−1^. The presence of prostate cancer leads to elevated PSA levels [4]. Therefore, there is an urgent need to develop effective methods to achieve sensitive detection of PSA [5,6,7,8]. To date, many techniques have been used for the quantitative detection of PSA, including enzyme-linked immunosorbent assay (ELISA), electrochemical technique, colorimetric technique, and fluorescence technique [9,10,11,12], but the complicated operation and unsatisfactory sensitivity of these methods have greatly hindered their wide application. Therefore, it is essential to explore a reliable, simple, rapid, and sensitive method for prostate-specific antigen detection.

Photoelectrochemical (PEC) sensing is a new sensing technology that uses a single-wavelength light source assembled with an electrochemical detection device [13,14]. Due to its ease of miniaturization and integration, PEC sensing offers higher sensitivity than conventional electrochemical sensing [15,16,17,18]. When a beam of light is irradiated on a semiconductor material and its energy is equal to or greater than the semiconductor band gap, electrons can jump from the valence band to the conduction band under the excitation of light and generate holes in the valence band, achieving the effective separation of electrons and holes [19,20]. After the separation of electrons and holes, a photovoltage is generated and a photocurrent is formed in the external circuit [21,22,23]. Using the interaction between the photoelectrode material and the substances, the quantitative analysis of PEC sensors can be achieved based on the relationship between the change in photocurrent or photovoltage and the concentration of biomarkers [24,25].

In the past few years, covalent organic frameworks (COFs), as one of the crystalline porous materials built from organic building blocks linked by covalent bonds, have attracted extensive attention due to their ordered structure with tunable functional groups, programmable pore size, and high overall porosity [26,27,28,29,30]. COFs can easily be endowed with photoactive properties by incorporating appropriate building blocks and linkages, making it possible to generate COF-based PEC sensors with good structural designation [31]. So far, only several COFs have been proposed in this field, such as D-TA COF film [32], TAPP-COF film [33], F-COF [34], and PAF-130 [35]. However, such PEC sensors are fabricated with bulk COFs and cannot meet the full requirements of certain applications [36]. The long conduction paths between adjacent layers make it difficult for the resulting electron hole pairs to separate quickly, which seriously affects the photoelectric activity. Compared with bulk COFs, two-dimensional (2D) covalent organic framework nanosheets (CONs) have unique properties, including tunable thicknesses down to 1.15 ± 0.1 nm (COF-367), large specific surface area, and highly accessible active sites. In addition, due to their extremely short layer spacing, ultrathin CONs have better photoelectric performance than that of bulk COFs, enabling effective separation of the generated electron–hole pairs.

The top-down approach can be used to prepare COF nanosheets. Soft and restorative structures were introduced into COF block for the fabrication of CONs [37,38]. As a type of 2D material composed of transition metals, carbides, nitrides, or carbonitrides, MXene possesses excellent electrical conductivity, large surface area, and tunable surface terminal groups, and is widely used in optoelectronic fields such as photocatalysis [39] and PEC sensors [40,41]. Since aluminum (Al) layers are replaced with various functional groups (e.g., -OH, -O, and -F) during etching, MXene can be easily integrated with other highly conductive materials or semiconductors to build composite systems [42,43]. As a semiconductor, the Fermi energy levels and the escape work of CONs and MXene are significantly different. This difference allows the generated electron-hole pairs to separate unidirectionally through the CONs/MXene interface, forming a space charge region that further prevents the passage of electrons or holes in the opposite direction. The composite is constructed to facilitate the separation and transport of electron-hole pairs while inhibiting their recombination, thus greatly improving the optoelectronic properties of the composite. Therefore, we prepared composite sensors by compounding polyethyleneimine (PEI)-modified CONs with Ti_3_C_2_T_x_ and applied them to the detection of PSA.

## 2. Results and Discussion

### 2.1. Morphology and Structure Characterization

**Scheme 1:**
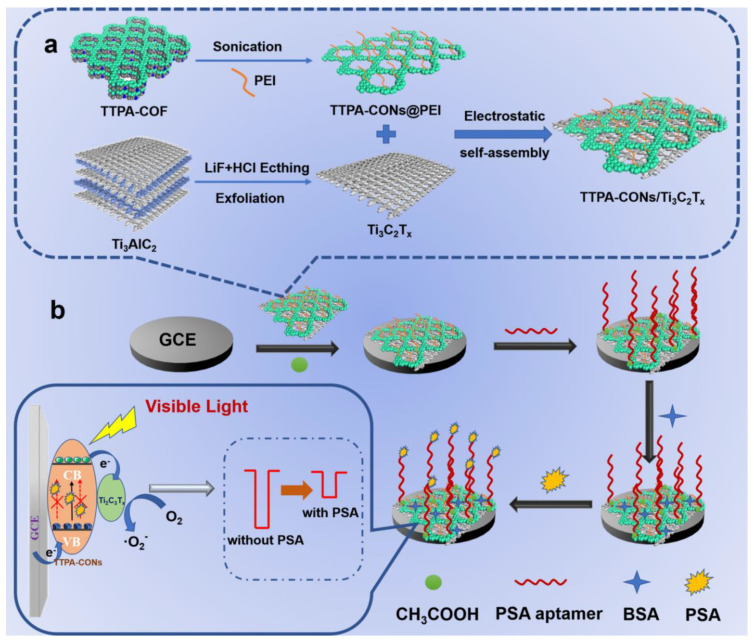
(**a**) Schematic illustration of TTPA-CONs/Ti_3_C_2_T_x_ composites prepared by electrostatic self-assembly. (**b**) TTPA-CONs/Ti_3_C_2_T_x_-based PEC sensor for PSA detection.

To realize the above design principle, it is necessary to obtain PEC sensors with hierarchical structures by electrostatic self-assembly. In our work, in order to obtain COF nanosheets directly by a bottom-up approach, we chose N,N,N′,N′-tetrakis(4-aminopheneyl)-1,4-phenylenediamine (TAPPDA) and N,N,N′,N′-tetrakis(4-formylpheneyl)-1,4-phenylenediamine (TFPPDA) with a distorted diarylamino structure to reduce the interlayer stacking of COF. After sonication, the TTPA-CONs were obtained. The as-synthesized TTPA-CONs were then electrostatically self-assembled with the newly synthesized Ti_3_C_2_T_x_ nanosheets (Scheme 1a). Typically, high yields of TTPA-COF were achieved by [4+4] imine condensation of TAPPDA and TFPPDA at a molar ratio of 1:1 in a mixture of *o*-dichlorobenzene (*o*-DCB) and N, N-dimethylacetamide (DMA), using 6 M acetic acid as the catalyst. To improve the yield of CONs, the obtained TTPA-COF was subjected to ultrasonication. Subsequently, the obtained TTPA-CONs were mixed with the freshly obtained Ti_3_C_2_T_x_ nanosheets. After sonication, TTPA-CONs/Ti_3_C_2_T_x_ composites were obtained (see the Materials and Methods section below for details). A typical C=N stretching vibration peak at ~1617 cm^−1^ can be observed in Fourier transform infrared spectroscopy (FT-IR). In addition, the disappearance of N-H stretching bonds in TAPA (3337 cm^−1^ and 3432 cm^−1^) and the reduction in C=O stretching bonds in TFPA (~1693 cm^−1^) indicate the formation of a Schiff base. Furthermore, TTPA-CONs exhibit FTIR spectra similar to those of TTPA-COF, indicating the retention of chemical structure after exfoliation (Figure S1).

**Figure 1:**
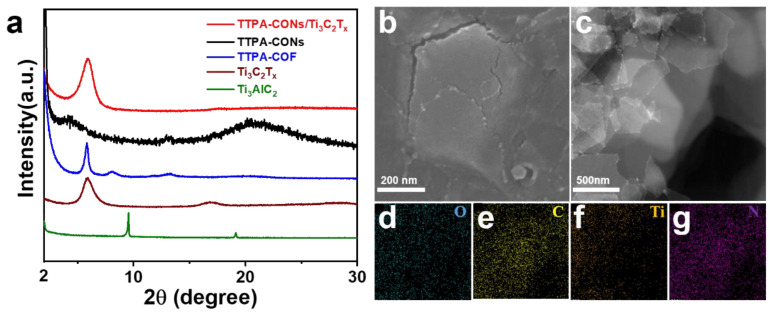
(**a**) PXRD diffraction patterns of TTPA-CONs/Ti_3_C_2_T_x_, TTPA-CONs, TTPA-COF, Ti_3_C_2_T_x_, and Ti_3_AlC_2_. (**b**) SEM, (**c**) TEM, and (**d**–**g**) elemental mapping of TTPA-CONs/Ti_3_C_2_T_x_ composites.

The crystal structures of the TTPA-CONs/Ti_3_C_2_T_x_ composites were further determined by power X-ray diffraction (PXRD) measurements and structural simulations (Figure 1a and Figure S2a,b). The experimental PXRD patterns of the obtained TTPA-COF match well with the simulated patterns of the crystal model. As shown in Figure S2a, a prominent diffraction peak arising from the (110) plane of TTPA-COF at 5.7° can be observed, as well as some weak peaks at 8.0° (200), 11.7° (220), and 13.0° (310), which is the same as our previous work [31]. After exfoliation, the main peak was extremely weakened and shifted to 4.0°. In addition, a weak and broad peak around 20.0° appears, indicating π–π stacking interaction between the vertically stacked 2D layers and the broadening of the layer distance of TTPA-COF, confirming the successful stripping of TTPA-COF (Figure 1a). Moreover, the disappearance of the typical diffraction peak of Al at 38.9° proves the successful etching of multilayer Ti_3_C_2_T_x_. In addition, the shift of the diffraction peak assigned to the (002) crystal plane from 9.5° to 6.2° originates from the removal of the aluminum atomic layer and the intercalation of lithium ions and H_2_O molecules after etching and exfoliation, which also represents the successful acquisition of Ti_3_C_2_T_x_ (Figure S2b). After electrostatic self-assembly, the composites show similar PXRD patterns to Ti_3_C_2_T_x_ without the characteristic peaks of TTPA-CONs, which could be attributed to the weaker peaks of TTPA-CONs compared with Ti_3_C_2_T_x_.

SEM images of the TTPA-CONs/Ti_3_C_2_T_x_ composites reveal the sheet-like morphology (Figure 1a and Figure S3a,b). The low contrast of TEM images corresponds to the ultrathin nature of the TTPA-CONs/Ti_3_C_2_T_x_ composites (Figure 1c and Figure S4a,b). As shown in Figure 1d–g, O, Ti, C, and N are uniformly distributed throughout the nanostructure, showing the homogeneity of TTPA-CONs and Ti_3_C_2_T_x_. To investigate the composite mode between TTPA-CONs and Ti_3_C_2_T_x_, we measured the zeta potential of TTPA-COF, TTPA-CONs, and Ti_3_C_2_T_x_. As shown in Figure S5, Ti_3_C_2_T_x_ exhibits a potential of −29.33 mV, which can be attributed to the presence of a large number of surface end groups (e.g., -O). Although TTPA-COF also exhibits a negative potential of -18.52 mV, the TTPA-CONs exhibit a positive potential of 65.13 mV after the functionalization of their exfoliated nanosheets with PEI. This suggests that TTPA-CONs and Ti_3_C_2_T_x_ can be bound by electrostatic adsorption.

**Figure 2:**
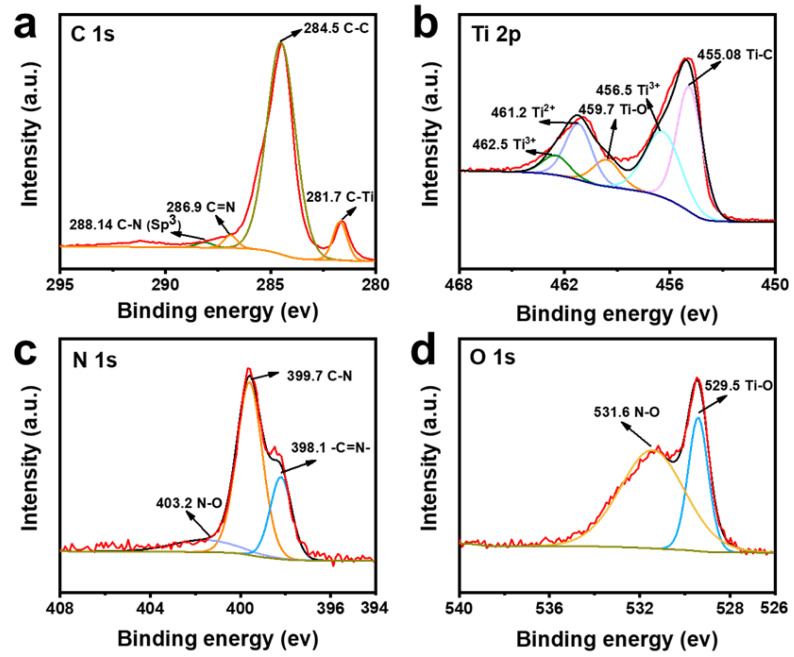
High-resolution C 1s (**a**), Ti 2p (**b**), N 1s (**c**), and O 1s (**d**) XPS spectra of TTPA-CONs/Ti_3_C_2_T_x_ composites.

X-ray photoelectron spectroscopy (XPS) measurements were used to further investigate the surface characteristics of the composites. The XPS survey spectra of TTPA-CONs/Ti_3_C_2_T_x_ in Figure S6 show the presence of N, O, Ti, and C elements. As shown in Figure 2a, the high-resolution C1s spectra of TTPA-CONs/Ti_3_C_2_T_x_ exhibit a series of typical peaks at 281.7 eV, 284.5 eV, 285.8 eV, 286.9 eV, and 288.14 eV, which can be assigned to C-Ti bonds, amorphous carbon, C-O bonds, C=N bonds, and C-N bonds, respectively, indicating the successful formation of TTPA-CONs. Figure 2b shows the high-resolution Ti 2p spectrum, providing five characteristic peaks located at 455.08 eV, 456.5 eV, 459.7 eV, 461.2 eV, and 462.5 eV, which can be ascribed to the corresponding bonds of Ti-C, Ti (iii), Ti-O, and Ti (ii) and Ti (iii), respectively. Among them, the presence of Ti-O indicates the implantation of functional groups (-O) on the surface of Ti_3_C_2_T_x_. In addition to peaks at 399.7 eV (C-N bond) and 398.1 eV (C=N bond), the N 1s spectrum obtained from CONs shows a peak at 403.2 eV, which can be attributed to the N-O bond (Figure 2c). The presence of this bond suggests that TTPA-CONs and Ti_3_C_2_T_x_ are bound by implanting functional groups O implanted on the surface of Ti_3_C_2_T_x_. The typical peak at 531.6 eV on the O 1s spectrum (N-O bond) also indicates a strong interfacial interaction between TTPA-CONs and Ti_3_C_2_T_x_ (Figure 2d).

### 2.2. Design of PEC Sensing Platform

**Figure 3:**
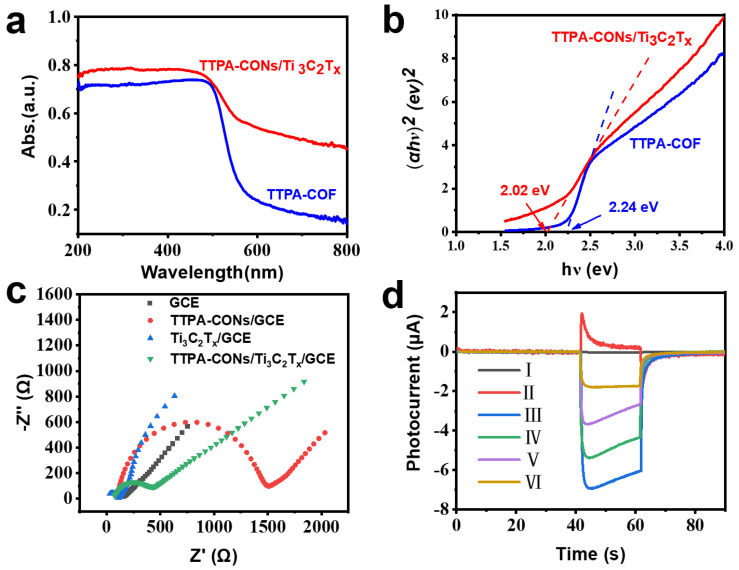
(**a**,**b**) UV–vis DRS spectra (**a**) and Tauc’s plots (**b**) of TTPA-COF and TTPA-CONs/Ti_3_C_2_T_x_. (**c**) EIS plots of GCE, TTPA-CONs/GCE, Ti_3_C_2_T_x_/GCE, and TTPA-CONs/Ti_3_C_2_T_x_/GCE. (**d**) Photocurrent responses of modified electrodes recorded at a bias potential of 0 V: (I) TTPA-CONs/GCE, (II) Ti_3_C_2_T_x_/GCE, (III) TTPA-CONs/Ti_3_C_2_T_x_/GCE, (IV) Aptamer/TTPA-CONs/Ti_3_C_2_T_x_/GCE, (V) BSA/Aptamer/TTPA-CONs/Ti_3_C_2_T_x_/GCE, and (VI) PSA/BSA/Aptamer/TTPA-CONs/Ti_3_C_2_T_x_/GCE. The PSA concentration used in (VI) was 10 ng/mL.

Figure 3a shows the UV–vis diffuse reflectance spectra of TTPA-COF and TTPA-CONs/Ti_3_C_2_Tx composites. The TTPA-COF sample reveals a well-established UV absorption spectrum with a shoulder absorption peak at 475 nm, while the TTPA-CONs/Ti_3_C_2_T_x_ composite material shows a distinct absorption at 490 nm. The broad absorption bands of TTPA-CONs/Ti_3_C_2_T_x_ and TTPA-COF in the visible region can be attributed to the distorted diarylamino units of TAPPDA and TFPPDA. Compared with pure TTPA-COF, the absorption capacity of TTPA-CONs/Ti_3_C_2_T_x_ composites (red line) is significantly enhanced. As depicted in Figure 3b, the band gap energies (*E_g_*) of TTPA-COF and TTPA-CONs/Ti_3_C_2_T_x_ composites are 2.24 eV and 2.02 eV, respectively. In addition, the fabrication steps of the various photoelectrodes were characterized. Electrochemical impedance spectrum (EIS) shows the interfacial behavior of the prepared electrodes in 5 mM K_3_[Fe(CN)_6_]**^−^** solution containing 0.1 M KCl (Figure 3c and Figure S7).

The diameter of the semicircle in the high-frequency region represents the charge transfer resistance (R_ct_). Accordingly, the semicircular diameter of TTPA-CONs/Ti_3_C_2_T_x_ composites is significantly smaller than that of the pristine TTPA-CONs, suggesting that the charge carriers in the TTPA-CONs/Ti_3_C_2_T_x_ composites have a smaller charge transfer resistance and faster transfer rate. In addition, Figure 3d depicts the transient cathodic PEC response of different photoelectrodes under visible light irradiation. The photocurrent intensities of TTPA-CONs and Ti_3_C_2_T_x_ show a small value (curve I, II), while the photocurrent intensity of TTPA-CONs/Ti_3_C_2_T_x_ is significantly higher, recorded as 6.9 μA (curve Ⅲ). As shown in Scheme 1b, light excites photogenerated electrons to the conduction band (CB) of TTPA-CONs, and these electrons can be transferred from the conduction band (CB) of TTPA-CONs to Ti_3_C_2_T_x_, which can effectively improve the efficiency of photoelectron–hole pair separation due to its excellent electron transport ability at the TTPA-CONs/Ti_3_C_2_T_x_ composites interface, and further suppress the photoelectron–hole pair complexation. The photogenerated electrons of Ti_3_C_2_T_x_ are trapped by molecular oxygen to produce active •O_2_^−^, while the photogenerated holes in the VB of TTPA-CONs are covered by electrons from the electrode to produce cathodic photocurrent [44]. Thus, the main reasons for obtaining the photoelectrochemical activity are the enhanced absorption of visible light and the high separation and transfer efficiency of the photogenerated carriers.

Afterward, the NH_2_-terminal aptamer was immobilized on the acetic acid-catalyzed TTPA-CONs/Ti_3_C_2_T_x_ surface due to the abundance of aldehyde groups on the surface of TTPA-CONs/Ti_3_C_2_T_x_, and the photocurrent intensity was measured to be 5.4 μA (curve Ⅳ). The decrease in the photocurrent density was due to the restart of electron transfer at the PEC sensing interface by the loading of the poorly conducting aptamer. After treatment with BSA, the photocurrent density was further reduced to 3.6 μA (curve Ⅴ). Finally, when the target PSA was trapped on the photoelectrode, the photocurrent density further decreased to 1.8 μA (curve Ⅵ). The reason for this decrease in photocurrent density may be related to the following reasons. PSA can be trapped at the sensing interface due to the biorecognition reaction between the aptamer and its target, while the formation of PSA-aptamer complexes with large steric hindrance increases the electron transfer resistance and adversely affects the photocurrent response. Therefore, significant changes in photocurrent density can be used for the quantitative detection of PSA.

### 2.3. Optimization of Experimental Conditions

In order to obtain better PEC performance, we investigated the effect of each experimental parameter on the photocurrent intensity. As shown in Figure S8, the photocurrent intensity increased with increasing concentration of TTPA-CONs/Ti_3_C_2_T_x_ in the range of 1 to 3.0 mg L^−1^. When the concentration of TTPA-CONs/Ti_3_C_2_T_x_ was further increased, the photocurrent intensity decreased rapidly, which may be due to the thicker nanocoating hindering electron transfer. Therefore, a TTPA-CONs/Ti_3_C_2_T_x_ concentration of 3.0 mg mL^−1^ was chosen for subsequent experiments. Next, we optimized the pH of the Tris-HCl solution. As shown in Figure S9, the photocurrent intensity gradually increased and then gradually decreased as the pH increased from 6.0 to 7.4, which indicated that the Tris-HCl solution with pH 7.4 was the most favorable for electron transfer, so we chose the solution with pH 7.4 to detect PSA. In addition, the incubation time of the PSA aptamer, BSA, and PSA had important effects on the performance of the PEC sensor. The photocurrent intensity decreased rapidly with increasing incubation time and reached the lowest values at 2.5 h, 50 min, and 120 min, respectively (Figures S10–S12). Subsequently, the photocurrent did not change greatly with the extension of time, so the incubation times for the PSA aptamer, BSA, and PSA were finally determined to be 2.5 h, 50 min, and 120 min, respectively. Since the PSA aptamer can bind to P4, which can directly affect the intensity of the photocurrent, the concentration of the P4 aptamer was optimized in Figure S13. The photocurrent intensity reached the highest value when the concentration of PSA aptamer was 5 μM. Finally, we chose the concentration of P4 aptamer to be 5 μM for subsequent experiments.

### 2.4. Analytical Behavior of the Proposed PEC Sensing Platform

**Figure 4:**
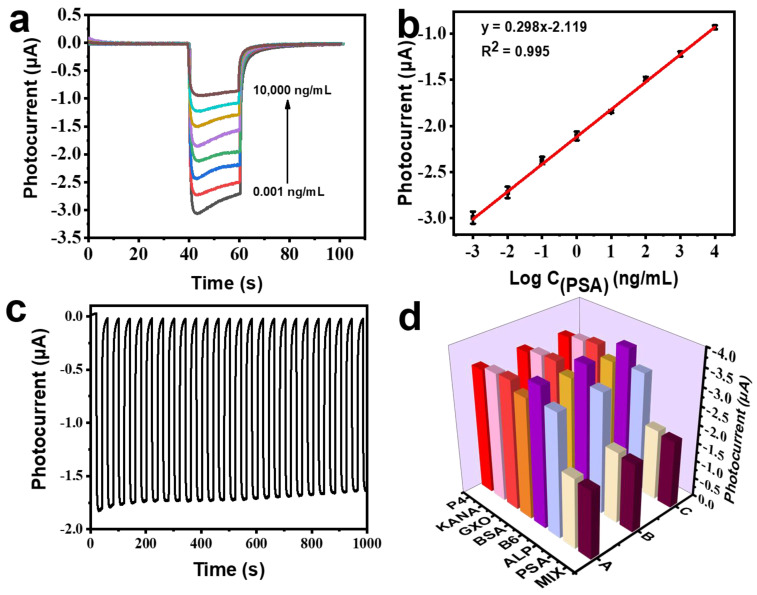
(**a**) Photocurrent responses of the fabricated PEC sensor after incubation with different concentrations of PSA in 0.1 M Tris-HCl (pH 7.4). (**b**) Calibration curve for PSA on the PEC sensor. (**c**) Stability of the photocurrent response in repeated on/off irradiation with an interval of 20 s. (**d**) Evaluation of the specificity of the PEC sensor for interfering substances. Note that P4, KANA, GXO, BSA, B6, ALP, and PSA represent the photocurrent intensities of fabricated PEC sensors with the addition of 10 μM P4, 10 μM KANA, 10 μM GXO, 10 μM BSA, 10 μM B6, 10 μM ALP, and 10 ng/mL PSA, in 0.1 M Tris-HCl buffer, respectively. MIX represents the photocurrent intensity of the fabricated PEC sensor with the addition of all the above substances. A, B, and C represent three sets of mutually independent experiments.

The photocurrent intensities of the fabricated PEC sensors with the addition of different concentrations of PSA were recorded under optimal conditions. As shown in Figure 4a, the photocurrent intensities gradually decreased with increasing concentrations of PSA in the range of 0.001–10,000 ng/mL. This can be explained by the fact that a large amount of poorly conducting PSA-aptamer complexes is generated by being trapped on the sensing surface, hindering electron transfer. As shown in Figure 4b, the logarithm of photocurrent intensity and PSA concentration showed a good linear relationship in the range of 0.001 to 10,000 ng/mL. The corresponding linear regression equation was *I* (μA) = 0.298 log *C*_PSA_ (ng/mL)–2.119 (correlation coefficient R^2^ = 0.995). In addition, the limit of detection (LOD) was estimated to be 0.0003 ng/mL based on the analytical function LOD = Kσ/S, where K is 3, σ is the standard deviation of the blank solution (*n* = 10), and S is the slope of the regression line. Compared with many previously reported sensors for the determination of PSA (Table S1), the PEC biosensor exhibited an acceptable linear range and low detection limit. The formation of the composite excited excellent photoelectrochemical activity, including intense visible light collection, effective suppression of the recombination rate of light-generated electron–hole pairs, and accelerated charge separation/transfer, leading to the high-performance of the proposed PEC sensor. On the other hand, the coupling of TTPA-CONs and Ti_3_C_2_T_x_ generates composites that minimize the mismatch between the interface and the lattice, contributing to the easy separation and transfer of photogenerated carriers. This feature is also an advantage for establishing an efficient PEC bioassay platform.

### 2.5. Selectivity, Stability, and Reproducibility of the PEC Sensor

The stability of the cathodic PEC conformal sensor was evaluated. Figure 4c shows that the photocurrent response of the PEC sensor remains stable under continuous illumination and 1000 s of repeated on/off illumination, showing significant stability. To investigate the interference of other hormones and metal ions on the assay, we performed interference measurements including Progesterone (P4), Kanamycin (KANA), glucose oxidase (GXO), Bovine Serum Albumin (BSA), Vitamin B6 (B6), and Alkaline phosphatase (ALP). As shown in Figure 4d, there is little change in the PEC response signal before and after the presence of the above-mentioned interferents. The photocurrent intensity was significantly reduced only in the presence of the target or in the coexistence of the target and the potential interferer. These results indicate that our constructed PEC platform has good selectivity for PSA detection.

To further evaluate the stability of this PEC sensor, we checked the reproducibility of the sensor by comparing five individual modified electrodes. As shown in Figure S14, the photocurrent response of the PEC sensor was tested in a PSA containing 10 ng/mL. All electrodes exhibited similar photocurrent intensities and the calculated relative standard deviation (RSD) was 2.79%. This indicates that the sensor has good repeatability and stability. In addition, the modified electrode was stored in a refrigerator at 4 °C and its photocurrent response was observed and measured every three days (Figure S15). The results showed that after 15 days of storage, the PEC sensor still maintained 95% of its initial response. This indicates that the PEC sensor has satisfactory storage stability.

To further evaluate the applicability of the prepared PEC sensor in real complex samples, we performed spiked recovery experiments on this PEC sensor. A cathodic PEC biosensor was used to determine the amount of PSA in bovine serum to simulate the amount of PSA in the prepared human serum. A certain concentration of standard PSA solution was added to the diluted serum sample, and the photocurrent response of the prepared electrode was recorded. As shown in Table S2, the recoveries ranged from 94.3% to 103.2% with RSD values less than 1.76%, demonstrating the feasibility and acceptable accuracy of the designed method for the determination of PSA in real samples.

## 3. Materials and Methods

N,N,N′,N′-tetrakis(4-aminopheneyl)-1,4-phenylenediamine (TAPPDA) and N,N,N′,N′-tetrakis(4-formylpheneyl)-1,4-phenylenediamine (TFPPDA) were synthesized according to the published method [45,46]. N, N-Dimethylformamide (DMF), polyethyleneimine (PEI) and N, N-Dimethylacetamide (DMA) were purchased from Shanghai Tensus Biotech Co., Ltd. 1,2-Dichlorobenzene (*o*-DCB, 99%), ethanol (EtOH) tetrahydrofuran (THF), acetone and acetic acid (AcOH, 99.5%) were purchased from Shanghai Macklin Biochemical Co., Ltd. Hydrochloric acid was purchased from Xilong Scientific Co., Ltd. Ti_3_AlC_2_ powder (400 mesh) was obtained from Laizhou Kai Xi Ceramic Materials Co., Ltd. Lithium fluoride (LiF) was received from Aladdin Reagent Co., Ltd. Progesterone (P4), glucose oxidase (GOX), Kanamycin (KANA), Vitamin B6 (B6), and Alkaline phosphatase (ALP), were acquired from Aladdin Bio-Chem Technology Co., Ltd. (Shanghai, China). Bovine Serum Albumin (BSA) was obtained from Shanghai Regal Biology Technology Co, Ltd. (Shanghai, China). The Aptamer of PSA (5′-NH_2_ C_6_ATTAAAGCTCGCCATCAAATAGC-3′) was purchased from Shanghai Sangon Biotechnology Co. Ltd. (Shanghai, China). The antigen of PSA was obtained from were all Shanghai Linc-Bio Science Co. LTD (Shanghai, China). Dilute the stock solution of the inducer with 10 mM phosphate buffer (PBS, pH 7.4) and store in the refrigerator freezer layer for further use. Ultra-pure water with a resistivity of 18.2 MΩ cm was used throughout the experiments.

Fourier transform infrared spectroscopy (FTIR) spectra were obtained with a Nicolet 6700 Thermo FT-IR spectrometer. Powder X-ray diffraction (PXRD) patterns were obtained on an X-ray powder diffractometer (D/max-Ultima IV) equipped with a Cu sealed tube (λ = 1.54178 Å) at a scan rate of 0.02 deg s^−1^. Scanning electron microscopy (SEM) was conducted on a FE-SEM (Nova NanoSEM 450) equipped with an energy dispersive spectrometer. Samples were treated via Pt sputtering before observation. Transmission electron microscopy (TEM) images were obtained using a transmission electron microscope (Talos S-FEG (FEI, Hillsboro, OR, USA)). X-ray photoelectron spectroscopy (XPS) analysis was measured on Thermo ESCALAB 250Xi spectrometer equipped with a light source of Al Ka X-ray (1486.6 eV) (Thermoelectricity Instruments, USA). UV-Vis diffuse reflectance spectroscopy (UV-Vis DRS) spectra were obtained by a UV-Vis spectrophotometer (UV-Vis-NIR Cary 5000) and the data were converted to Kubelka-Munk functions for the band gap extraction. PEC measurements were carried out on a CHI760 E electrochemical workstation (Shanghai Chenhua Instrument Co., Shanghai, China) equipped with PLS-FX300HU xenon lamp parallel light source system (Beijing Perfect light Technology Co., Ltd., Beijing, China).

### 3.1. Synthesis of TTPA-COF

A 10 mL glass vial was charged with N,N,N′,N′-tetrakis(4-formylpheneyl)-1,4-phenylenediamine (TFPPDA) (11 mg, 0.2 mmol), N,N,N′,N′-tetrakis(4-aminopheneyl)-1,4-phenylenediamine (TAPPDA) (10 mg, 0.2 mmol) and N, N-Dimethylacetamide (DMA)/1,2-Dichlorobenzene (o-DCB) (*v*:*v* = 1/3, 1.2 mL). The mixture was sonicated for 10 min to get a homogenous dispersed russet solution. Subsequently, acetic acid (6 M, 0.12 mL) was added and the vial was then flash frozen at 77 K using a liquid N_2_ bath and degassed by three freeze-pump-thaw cycles. Subsequently, the tube was sealed under vacuum, and then heated at 120 °C for 3 days. The yielded orange precipitate was collected by centrifugation and immersed with N, N-Dimethylformamide (DMF) for 6 h under 80 °C by 2 cycles, separately. The collected powder was then activated by solvent exchange with anhydrous tetrahydrofuran (THF) and anhydrous acetone in Soxhlet extractor for 2 days, and dried at 80 °C under vacuum for 12 h to give an orange powder with 85 % isolated yield.

### 3.2. Preparation of TTPA-CONs

A 200 mL glass vial was charged with TTPA-COF (10 mg) and 100 mL DI water. Then 2 mL polyethyleneimine (PEI) solution (30 wt %, M.W. 70,000) was added and stirred at room temperature for 0.5 h. Subsequently, the mixture was sonicated for 8 h. After sonication, the dispersion was centrifuged at 3500 rpm for 10 min to obtain the supernatant. Then the obtained supernatant was centrifuged at 10,000 rpm for 10min to obtain TTPA-CONs, which was subsequently re-dispersed in DI water with a concentration of 500 μg /L.

### 3.3. Preparation of Ti_3_C_2_T_x_

Ti_3_C_2_T_x_ was prepared according to reported methods [40,41]. Specifically, LiF (0.5 g) was dissolved in 10 mL HCl (9 M) under room temperature. Subsequently, 0.5g Ti_3_AlC_2_ powder was slowly added within 5min and stirred at 35 °C for 24 h. After reaction, the suspension was washed with DI water by centrifugation repeatedly until the pH of the supernatant was greater than 6.0 was ultrasonic dispersed in DI water for 20 min under N_2_ atmosphere for 20 min. Finally, the supernatant containing Ti_3_C_2_T_x_ nanosheets (10 mg/mL) was collected by centrifugation at 7500 rpm for 20 min. The powder with ultra-thin and low-layer Ti_3_C_2_T_x_ nanosheets was obtained by freeze-dried for yield measurement.

### 3.4. Preparation of TTPA-CONs/Ti_3_C_2_T_x_

The TTPA-CONs/Ti_3_C_2_T_x_ composite material was synthesized by electrostatic self-assembly. Specifically, the as-obtained Ti_3_C_2_T_x_ suspension (50 μL) was diluted with DI water to 1 mL. Then, the Ti_3_C_2_T_x_ suspension was mixed with 1 mL TTPA-CONs suspension. The TTPA-CONs/Ti_3_C_2_T_x_ composite materials was obtained after sonicated for 10 min.

### 3.5. Fabrication of the PEC sensor

Scheme 1 illustrates the fabrication of the PEC biosensor based on the TTPA-CONs/Ti_3_C_2_T_x_ composites for PSA detection. Prior to modification, the glassy carbon electrodes (GCE) were polished with 0.3 μm and 0.05 μm alumina slurry and sonicated in ethanol and ultrapure water. Then, the TTPA-CONs/Ti_3_C_2_T_x_ composites were sonicated in ultrapure water for 1 min to obtain a uniformly distributed suspension with a concentration of 3 mg mL^−1^. The mixture was activated at room temperature with the 100 μL of 3M acetic acid added to 2 mL of 3 mg mL^−1^ TTPA-CONs/Ti_3_C_2_T_x_ solution and dispersed well. 10 μL of the above solution was dropped on GCE and evaporated at room temperature, and the modified photoelectrodes were named as TTPA-CONs/Ti_3_C_2_T_x_/GCE. The modified electrodes were defined as the Apt/TTPA-CONs/Ti_3_C_2_T_x_/GCE electrodes by incubating Aptamer-NH_2_ (5 μM, 10 μL) on TTPA-CONs/Ti_3_C_2_T_x_/GCE for 2.5 h at 4 °C. The modified electrodes were defined as BSA/Apt/TTPA-CONs/Ti_3_C_2_T_x_/GCE by incubation with 10 μL of 3 wt % BSA at 37 °C for 50 min to block the non-specific binding sites, followed by careful washing with ultrapure water. Next, the PSA series solutions with different concentrations were incubated at 4 °C for 120 min, and then washed for further measurements. PEC experiments were recorded on a CHI760 E electrochemical workstation using a three-electrode system with modified GCE as working electrode, platinum wire as counter electrode and Ag/AgCl as reference electrode. The photocurrent response was measured in 0.1 M phosphate buffer (pH 7.4) under visible light irradiation (300 W Xenon lamp) at room temperature without applying bias potential.

## 4. Conclusions

In summary, we herein demonstrated a novel PEC sensor based on composites constructed by electrostatic self-assembly of TTPA-CONs and Ti_3_C_2_T_x_. The prepared TTPA-CONs/Ti_3_C_2_T_x_ composites show enhanced photoelectric properties compared with TTPA-CONs, making them effective candidates for fabricating cathodic PEC sensors. Due to the effective separation of photogenerated electrons and holes, the well-designed PEC sensor exhibits a linear response to PSA in the range of 0.001 to 10,000 ng/mL, with a low detection limit of 0.0003 ng/mL under optimal conditions. It is believed that this work may pave the way toward the design of high-performance PEC sensing platforms and broaden the application of CONs in biomimetic sensing and analysis.

## Data Availability

Data can be found in the manuscript and in the Supplementary Materials.

## Supplementary Materials

The following supporting information can be downloaded at: https://www.mdpi.com/article/10.3390/molecules27196732/s1. Figure S1 (FT-IR spectra of TTPA-CONs, TTPA-COF, TFPA, and TAPA), Figure S2 (Experimental and simulated PXRD diffraction patterns of TTPA-COF and Ti_3_C_2_T_x_), Figure S3 (SEM images of TPA-CONs/Ti_3_C_2_T_x_), Figure S4 (TEM images of TPA-CONs/Ti_3_C_2_T_x_), Figure S5 (Zeta potential measurements of TTPA-COF, TTPA-CONs, and Ti_3_C_2_T_x_), Figure S6 (XPS spectrum of TPA-CONs/Ti_3_C_2_T_x_), Figure S7 (EIS plots of GCE, TTPA-CONs/GCE, Ti_3_C_2_T_x_/GCE, and TTPA-CONs/Ti_3_C_2_T_x_/GCE) and Figure S8–S15 (PEC performance of fabricated sensors) are available in supplementary information [47,48,49,50,51,52,53,54].

## Sample Availability

The compounds reported in this work are commercially available or can be synthesized according to the methods reported in this work.

## References

[1] Wang X., Xu R., Sun X., Wang Y., Ren X., Du B., Wu D., Wei Q. (2017). Using reduced graphene oxide-Ca: CdSe nanocomposite to enhance photoelectrochemical activity of gold nanoparticles functionalized tungsten oxide for highly sensitive prostate specific antigen detection. Biosens. Bioelectron..

[2] Huang L., Chen J., Yu Z., Tang D. (2020). Self-powered temperature sensor with seebeck effect transduction for photothermal–thermoelectric coupled immunoassay. Anal. Chem..

[3] Lv S., Zhang K., Zhu L., Tang D. (2019). ZIF-8-assisted NaYF4: Yb Tm@ ZnO converter with exonuclease III-powered DNA walker for near-infrared light responsive biosensor. Anal. Chem..

[4] Wu J., Fu Z., Yan F., Ju H. (2007). Biomedical and clinical applications of immunoassays and immunosensors for tumor markers. Trends Anal. Chem..

[5] Lance R., Drake R. (2011). Dean A Multiple recognition assay reveals prostasomes as promising plasma biomarkers for prostate cancer. Expert Rev. Anticancer Ther..

[6] Allison R., Jeferson Y., José E., José G., Lima N., de Sousa P.G., Maria V., dos Santos José C. (2022). The Chemistry and Applications of Metal-Organic Frameworks (MOFs) as Industrial Enzyme Immobilization Systems. Melcucules.

[7] Souada M., Piro B., Reisberg S., Anquetin G., Noël V., Pham M. (2015). Label-free electrochemical detection of prostate-specific antigen based on nucleic acid aptamer. Biosens. Bioelectron..

[8] Jarju J., Lavender A., Begoña E., Vanesa R., Laura M. (2020). Covalent Organic Framework Composites: Synthesis and Analytical Applications. Melcucules.

[9] Fenner A. (2012). Novel “inverse sensitivity” enzyme-linked crystal-growth assay to detect ultralow PSA levels. Nat. Rev. Urol..

[10] Kim D., Lee N., Park J., Park I., Kim J., Cho H. (2010). Organic electrochemical transistor based immunosensor for prostate specific antigen (PSA) detection using gold nanoparticles for signal amplification. Biosens. Bioelectron..

[11] Xu J., Cao P., Fan Z., Luo X., Yang G., Qu T., Gao J. (2022). Rapid Screening of Lipase Inhibitors in Scutellaria baicalensis by Using Porcine Pancreatic Lipase Immobilized on Magnetic Core–Shell Metal–Organic Frameworks. Melcucules.

[12] Wang X., Zhao M., Nolte D. (2011). Prostate specific antigen detection in patient sera by fluorescence-free BioCD protein array. Biosens. Bioelectron..

[13] Zhang S., Liu D., Wang G. (2022). Covalent Organic Frameworks for Chemical and Biological Sensing. Melcucules.

[14] Zhao W., Xu J., Chen H. (2014). Photoelectrochemical DNA biosensors. Chem. Rev..

[15] Zhu Y., Xu Z., Gao J., Ji W., Zhang J. (2020). An antibody-aptamer sandwich cathodic photoelectrochemical biosensor for the detection of progesterone. Biosens. Bioelectron..

[16] Gao Y., Qi H., Shang M., Zhang J., Yan J., Song W. (2019). Carbon dots-sensitized amorphous MoSx photoanode: Sequential electrodeposition preparation and dual amplified photoelectrochemical aptasensing of adenosine. Biosens. Bioelectron..

[17] Sun B., Zhang K., Chen L., Guo L., Ai S. (2013). A novel photoelectrochemical sensor based on PPIX-functionalized WO_3_-rGO nanohybrid-decorated ITO electrode for detecting cysteine. Biosens. Bioelectron..

[18] Sun G., Wang P., Ge S., Ge L., Yu J., Yan M. (2014). Photoelectrochemical sensor for pentachlorophenol on microfluidic paper-based analytical device based on the molecular imprinting technique. Biosens. Bioelectron..

[19] Fan D., Bao C., Khan M., Wang C., Zhang Y., Liu Q., Zhang X., Wei Q. (2018). A novel label-free photoelectrochemical sensor based on NS-GQDs and CdS co-sensitized hierarchical Zn_2_SnO_4_ cube for detection of cardiac troponin I. Biosens. Bioelectron..

[20] Han F., Song Z., Nawaz M., Dai M., Han D., Han L., Fan Y., Xu J., Han D., Niu L. (2019). MoS_2_/ZnO-Heterostructures-Based Label-Free Visible-Light-Excited Photoelectrochemical Sensor for Sensitive and Selective Determination of Synthetic Antioxidant Propyl Gallate. Anal. Chem..

[21] Gao B., Zhao X., Liang Z., Wu Z., Wang W., Han D., Niu L. (2021). CdS/TiO_2_ Nanocomposite-Based Photoelectrochemical Sensor for a Sensitive Determination of Nitrite in Principle of Etching Reaction. Anal. Chem..

[22] Dai Z., Han N., Xiong M., Han X., Zuo Y., Wang K. (2020). Portable Photoelectrochromic Visualization Sensor for Detection of Chemical Oxygen Demand. Anal. Chem..

[23] Song Z., Fan G., Li Z., Gao F., Luo X. (2018). Universal Design of Selectivity-Enhanced Photoelectrochemical Enzyme Sensor: Integrating Photoanode with Biocathode. Anal. Chem..

[24] Bai X., Zhang Y., Gao W., Zhao D., Yang D., Jia N. (2020). Hollow ZnS-CdS nanocage based photoelectrochemical sensor combined with molecularly imprinting technology for sensitive detection of oxytetracycline. Biosens. Bioelectron..

[25] Li H., Li Y., Li J., Yang F., Xu L., Wang W., Yao X., Yin Y. (2020). Magnetic–Optical Core–Shell Nanostructures for Highly Selective Photoelectrochemical Aptasensing. Anal. Chem..

[26] Li M., Chen C., Shi Y., Li L. (2016). Heterostructures based on two-dimensional layered materials and their potential applications. Mater. Today.

[27] Kim S., Lim H., Lee J., Choi H. (2018). Synthesis of a scalable two-dimensional covalent organic framework by the photon-assisted imine condensation reaction on the water surface. Langmuir.

[28] Gupta A., Sakthivel T., Seal S. (2015). Recent development in 2D materials beyond graphene. Prog. Mater. Sci..

[29] Zhao M., Huang Y., Peng Y., Huang Z., Ma Q., Zhang H. (2018). Two-dimensional metal–organic framework nanosheets: Synthesis and applications. Chem. Soc. Rev..

[30] Dong R., Zhang T., Feng X. (2018). Interface-assisted synthesis of 2D materials: Trend and challenges. Chem. Rev..

[31] Peng Y., Huang M., Chen L., Gong C., Li N., Huang Y., Cheng C. (2022). Ultrathin covalent organic framework nanosheet-based photoregulated metal-free oxidase-like nanozyme. Nano Res..

[32] Liu T., Cui L., Zhao H., Zhang X. (2020). In situ generation of regularly ordered 2D ultrathin covalent organic framework films for highly sensitive photoelectrochemical bioanalysis. ACS Appl. Mater. Interfaces.

[33] Zhao C., Zhang L., Wang Q., Zhang L., Zhu P., Yu J., Zhang Y. (2021). Porphyrin-based covalent organic framework thin films as cathodic materials for “on-off-on” photoelectrochemical sensing of lead ions. ACS Appl. Mater. Interfaces.

[34] Wang C., Liu N., Liu X., Tian Y., Zhai X., Chen X., Hou B. (2021). Fluoro-Substituted Covalent Organic Framework Particles Anchored on TiO_2_ Nanotube Arrays for Photoelectrochemical Determination of Dopamine. ACS Appl. Nano Mater..

[35] Gao Y., Zhang J., Zhang X., Li J., Zhang R., Song W. (2021). Liposomal Controlled Release Ag-Activated DNA zyme Cycle Amplification on a 2D Pyrene COF-Based Photocathode for α-Synuclein Immunosensing. Anal. Chem..

[36] Chen L., Huang M., Chen B., Gong C., Li N., Cheng H., Chen Y., Peng Y., Xu G. (2021). Two-dimensional covalent organic framework nanosheets: Synthesis and energy-related applications. Chin. Chem. Lett..

[37] Wang H., Zeng Z., Xu P., Li L., Zeng G., Xiao R., Tang Z., Huang D., Tang L., Lai C. (2019). Recent progress in covalent organic framework thin films: Fabrications applications and perspectives. Chem. Soc. Rev..

[38] Guan Q., Wang G., Zhou L., Li W., Dong Y. (2020). Nanoscale covalent organic frameworks as theranostic platforms for oncotherapy: Synthesis functionalization and applications. Nanoscale Adv..

[39] Low J., Zhan L., Tong T., Shen B., Yu J. (2018). TiO_2_/MXene Ti_3_C_2_ composite with excellent photocatalytic CO2 reduction activity. J. Catal..

[40] Ye C., Wu Z., Wu K., Xia Z., Pan J., Wang M., Ye C. (2021). Ti_3_C_2_ MXene-based Schottky photocathode for enhanced photoelectrochemical sensing. J. Alloys Compd..

[41] Ye C., Xu F., Ullah F., Wang M. (2022). CdS/Ti_3_C_2_ heterostructure–based photoelectrochemical platform for sensitive and selective detection of trace amount of Cu^2+^. Anal. Bioanal. Chem..

[42] Khan A., Tahir M., Bafaqeer A. (2020). Constructing a stable 2D layered Ti_3_C_2_ MXene cocatalyst-assisted TiO_2_/g-C_3_N_4_/Ti_3_C_2_ heterojunction for tailoring photocatalytic bireforming of methane under visible light. Energy Fuels.

[43] Junaidi N., Wong W., Loh K., Rahman S., Daud W. (2021). A comprehensive review of MXenes as catalyst supports for the oxygen reduction reaction in fuel cells. Int. J. Energy Res..

[44] Zhang J., Gao Y., Liu P., Yan J., Zhang X., Xing Y., Song W. (2021). Charge transfer accelerated by internal electric field of MoS_2_ QDs-BiOI pn heterojunction for high performance cathodic PEC aptasensing. Electrochim. Acta.

[45] El-Mahdy A., Mohamed M., Mansoure T., Yu H., Chen T., Kuo S. (2019). Ultrastable tetraphenyl-pphenylenediamine-based covalent organic frameworks as platforms for high-performance electrochemical supercapacitors. Chem. Commun..

[46] Ito A., Kurata R., Sakamaki D., Yano S., Kono Y., Nakano Y., Furukawa K., Kato T., Tanaka K. (2013). Redox modulation of *para*-phenylenediamine by substituted nitronyl nitroxide groups and their spin states. J. Phys. Chem..

[47] Yang C., Guo Q., Lu Y., Zhang B., Nie G. (2020). Ultrasensitive "signal-on" electrochemiluminescence immunosensor for prostate-specific antigen detection based on novel nanoprobe and poly (indole-6-carboxylic acid)/flower-like Au nanocomposite. Sens. Actuators B Chem..

[48] Zhao Y., Zheng F., Shi L., Liu H., Ke W. (2019). Autoluminescence-Free Prostate-Specific Antigen Detection by Persistent Luminous Nanorods and Au@Ag@SiO2 Nanoparticles. ACS Appl. Mater. Interfaces.

[49] Zhao Y., Cui L., Sun Y., Zheng F., Ke W. (2019). Ag/CdO NP-Engineered Magnetic Electrochemical Aptasensor for Prostatic Specific Antigen Detection. ACS Appl. Mater. Interfaces.

[50] Fan D., Li N., Ma H., Li Y., Hu L., Du B., Wei Q. (2016). Electrochemical immunosensor for detection of prostate specific antigen based on an acid cleavable linker into MSN-based controlled release system. Biosens. Bioelectron..

[51] Dai L., Li Y., Wang Y., Luo X., Wei D., Feng R., Yan T., Ren X., Du B., Wei Q. (2019). A prostate-specific antigen electrochemical immunosensor based on Pd NPs functionalized electroactive Co-MOF signal amplification strategy. Biosens. Bioelectron..

[52] Cao J., Dong Y., Ma Y., Wang B., Ma S., Liu Y. (2020). A ternary CdS@Au-g-C3N4 heterojunction-based photoelectrochemical immunosensor for prostate specific antigen detection using graphene oxide-CuS as tags for signal amplification. Anal. Chim. Acta..

[53] Zhao J., Wang S., Zhang S., Zhao P., Wang J., Yan M., Ge S., Yu J. (2020). Peptide cleavage-mediated photoelectrochemical signal on-off via CuS electronic extinguisher for PSA detection. Biosens. Bioelectron..

[54] Sun X., Li C., Zhu Q., Chen J., Li J., Ding H., Sang F., Kong L., Chen Z., Wei Q. (2019). A novel ultrasensitive sandwich-type photoelectrochemical immunoassay for PSA detection based on dual inhibition effect of Au/MWCNTs nanohybrids on N-GQDs/CdS QDs dual sensitized urchin-like TiO2. Electrochim. Acta.

