# Supplementary material for: Ultrathin Covalent Organic Framework Nanosheets/Ti3C2Tx-Based Photoelectrochemical Biosensor for Efficient Detection of Prostate-Specific Antigen
Source: Molecules. 2022 Oct 9;27(19):6732. doi: 10.3390/molecules27196732 (PMC9572316; doi:10.3390/molecules27196732)
Supplement: Supplementary file 1 [file molecules-27-06732-s001.zip › molecules-1934794-supplementary.pdf]

Supporting Information for

Ultrathin Covalent Organic Framework Nanosheets/$Ti_3C_2T_x$-Based Photoelectrochemical Biosensor for Efficient Detection of Prostate-specific Antigen

Nanjun Li[†], Chongyang Wang[†], Liangjun Chen, Cui Ye*, and Yongwu Peng*

College of Materials Science and Engineering, Zhejiang University of Technology, Hangzhou 310014, China

[†]These authors contributed equally to this work

*Corresponding author, ywpeng@zjut.edu.cn; ye0702@zjut.edu.cn

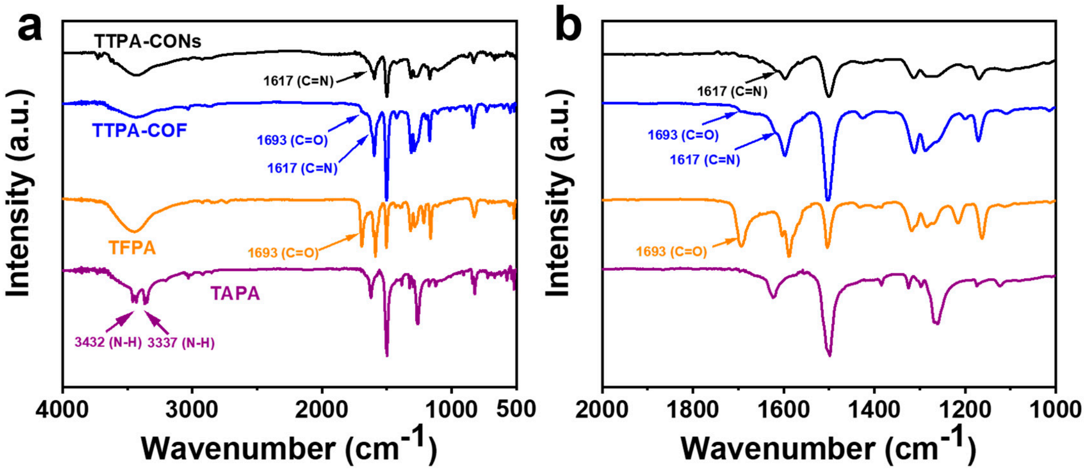

Figure S1. (a) FT-IR spectra of TTPA-CONs, TTPA-COF, TFPA, and TAPA. (b) Enlarged FT-IR spectra of figure S1a.

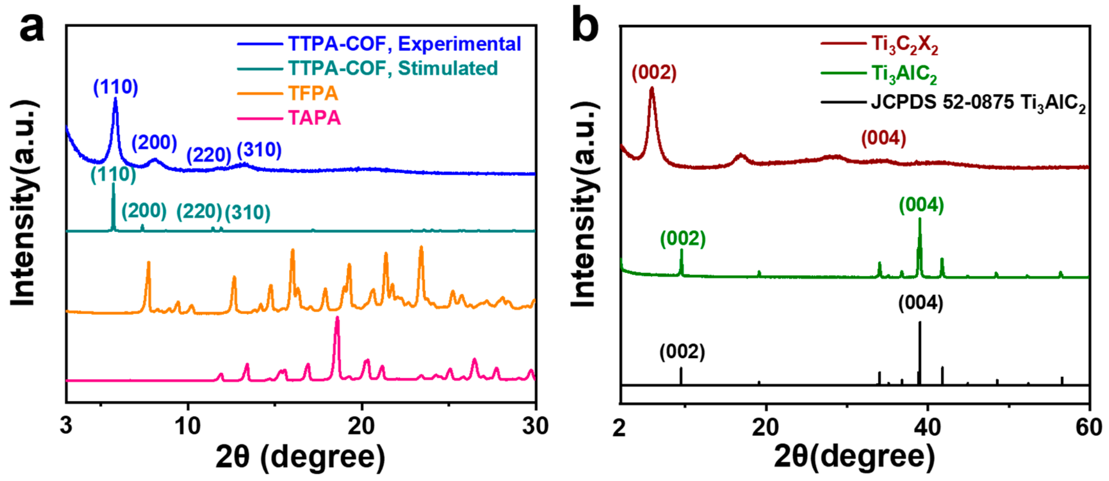

Figure S2. (a) Experimental and simulated PXRD patterns of TTPA-COF, and PXRD patterns of TFPA, TAPA. (b) PXRD patterns of $Ti_3C_2T_x$ and Ti_3AlC_2.

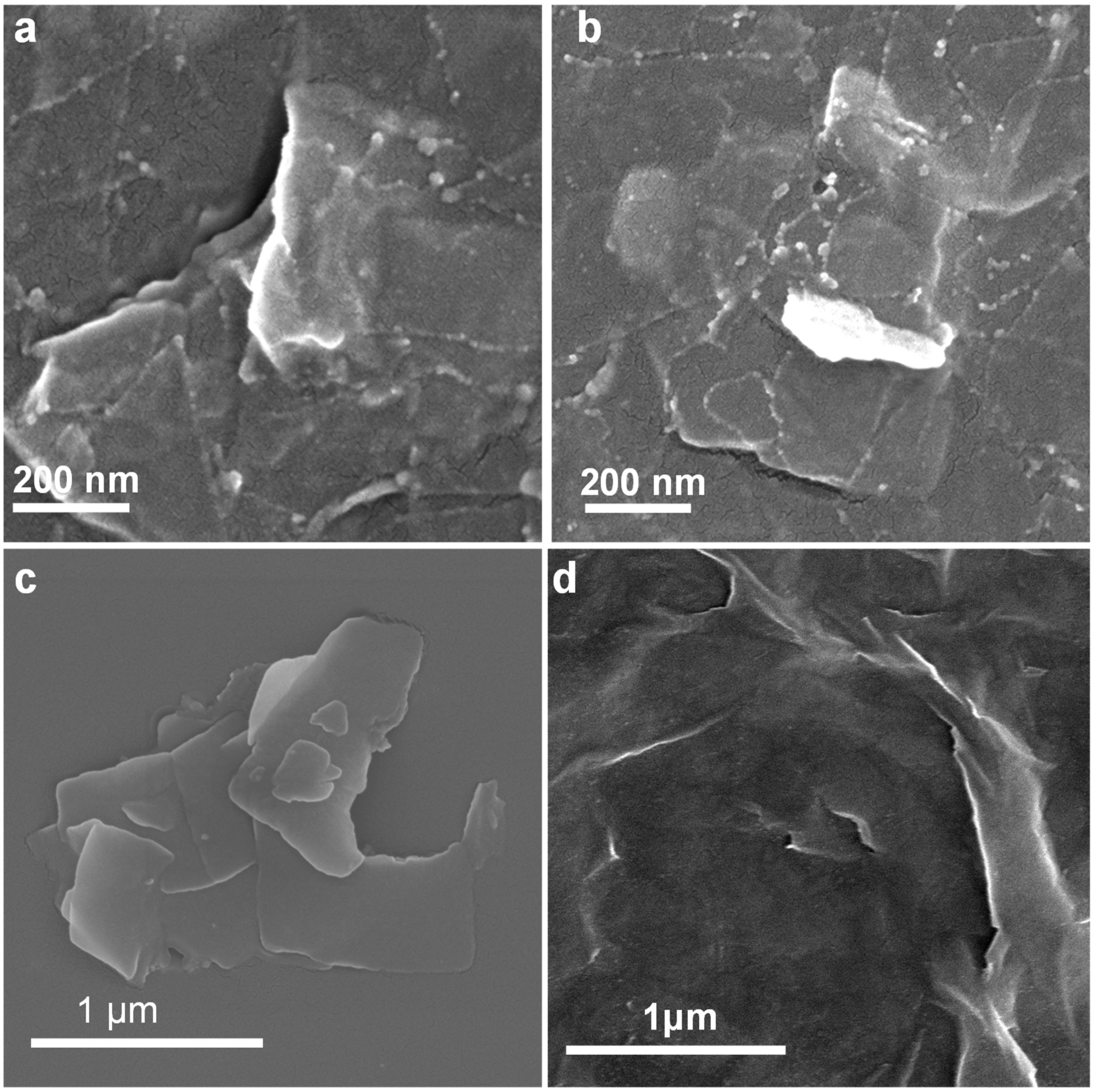

Figure S3. SEM images of TPA-CONs/$Ti_3C_2T_x$ (a, b), TPA-CONs (c), and $Ti_3C_2T_x$ (d).

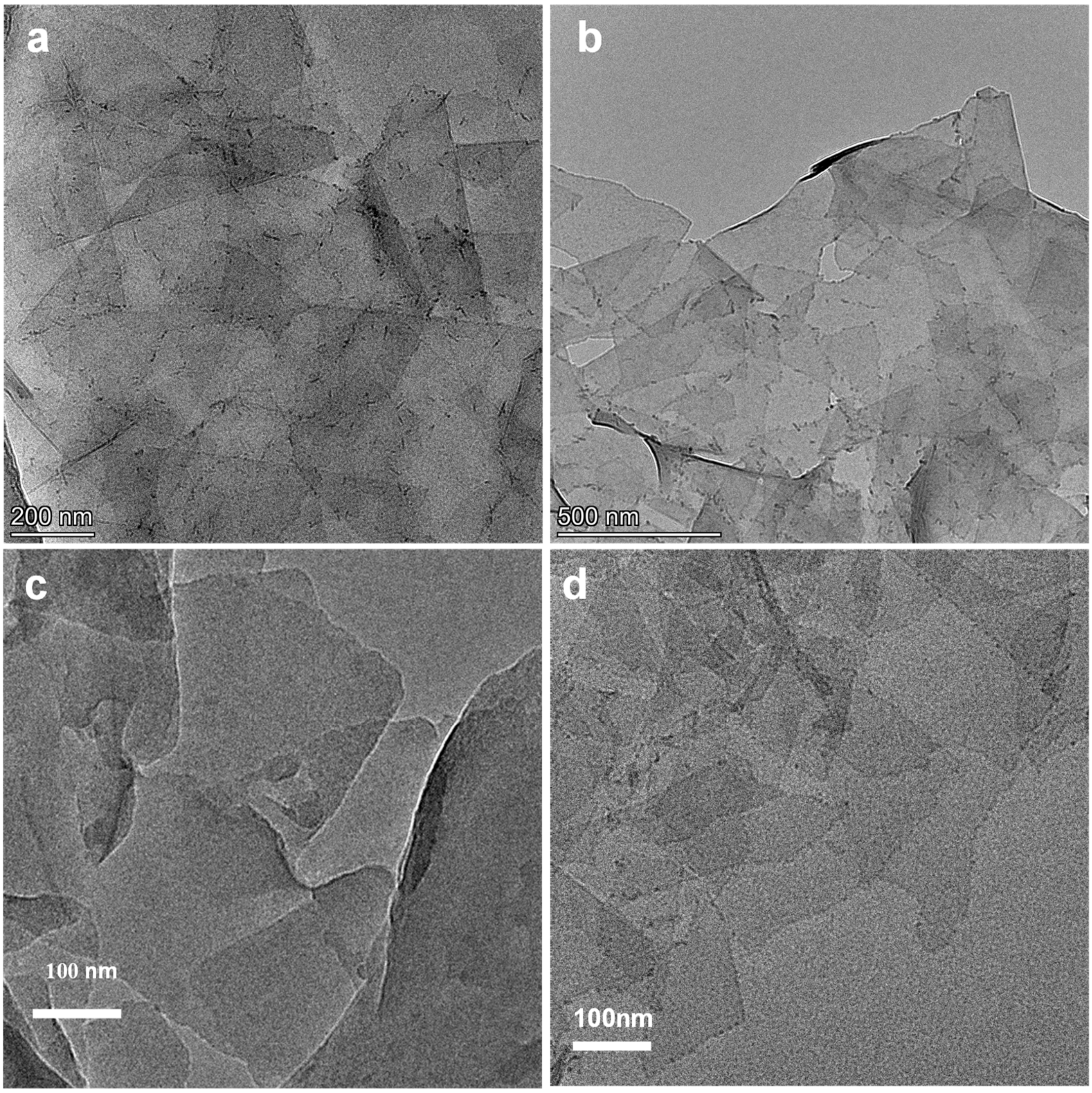

Figure S4. TEM images of TPA-CONs/$Ti_3C_2T_x$ (a, b), TPA-CONs (c), and $Ti_3C_2T_x$ (d).

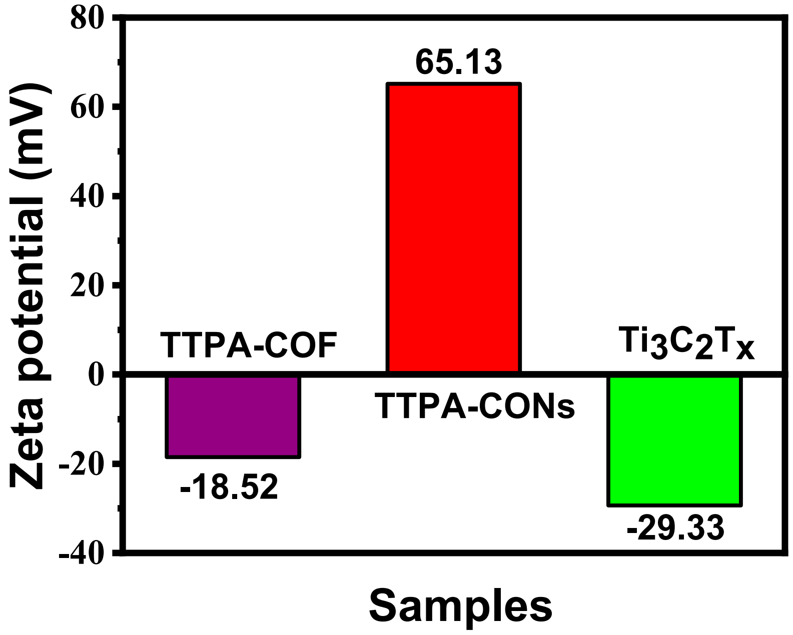

Figure S5. Zeta potential measurements of TTPA-COF, TTPA-CONs, and $Ti_3C_2T_x$.

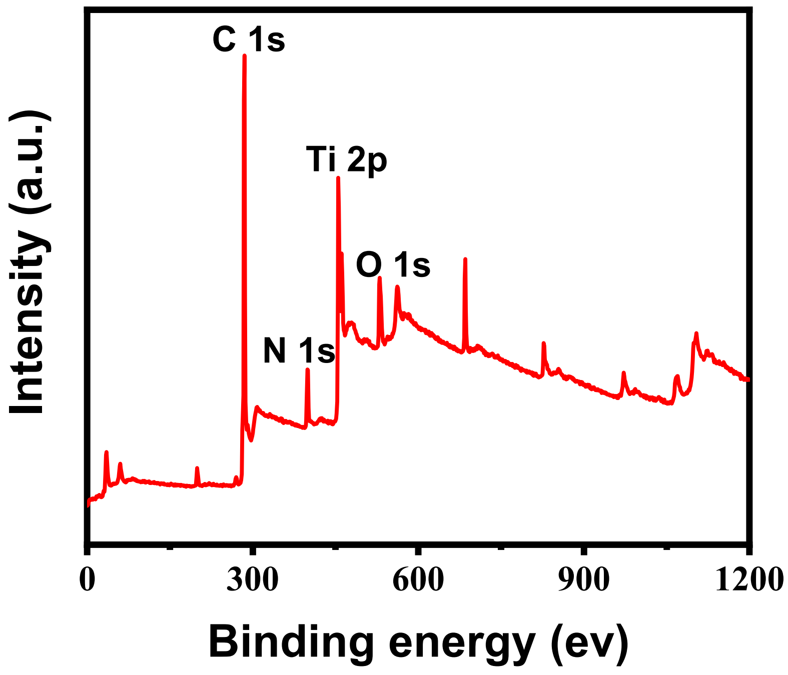

Figure S6. XPS spectrum of TTPA-CONs/$Ti_3C_2T_x$.

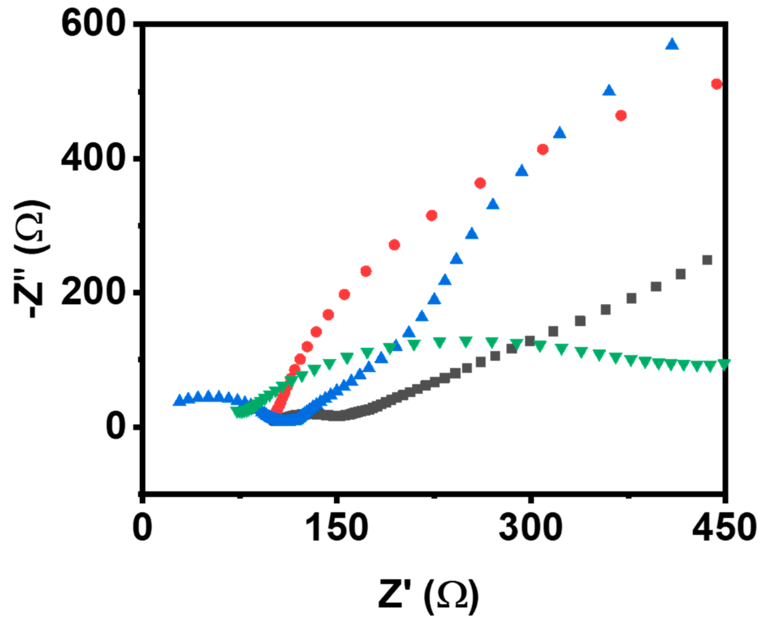

Figure S7. EIS plots of GCE, TTPA-CONs/GCE, $Ti_3C_2T_x$/GCE, and TTPA-CONs/$Ti_3C_2T_x$/GCE.

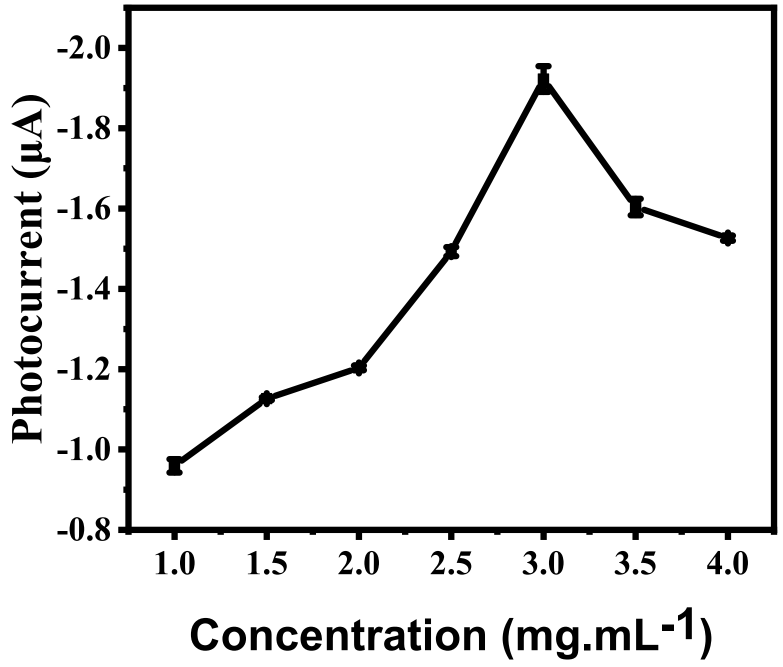

Figure S8. Relationship between photocurrent intensity and concentration of TTPA-CONs/$Ti_3C_2T_x$. Aptamer concentration: 5 μM. Incubation time: 2.5 h. BSA content: 3 wt%. Incubation time: 50 min. PSA concentration: 10 ng/mL. Incubation time: 120 min. pH of the buffer solution: 7.4. Error bars are derived from the standard deviation of three measurements.

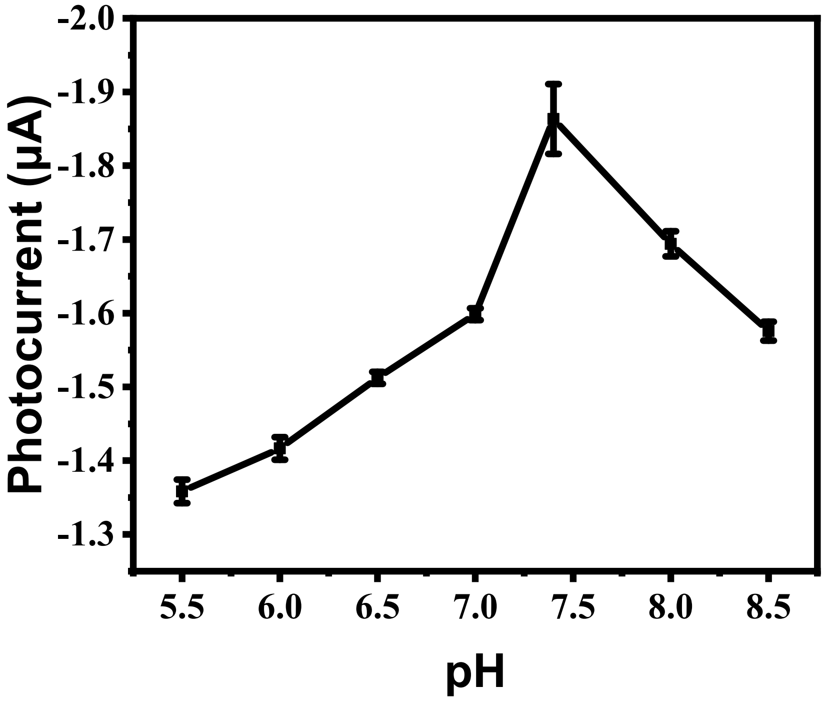

Figure S9. Relationship between photocurrent intensity and pH of the buffer solution. TTPA-CONs/$Ti_3C_2T_x$ concentration: 3 mg/mL. Aptamer concentration: 5 μM. Incubation time: 2.5 h. BSA content: 3 wt%. Incubation time: 50 min. PSA concentration: 10 ng/mL. Incubation time: 120 min. Error bars are derived from the standard deviation of three measurements.

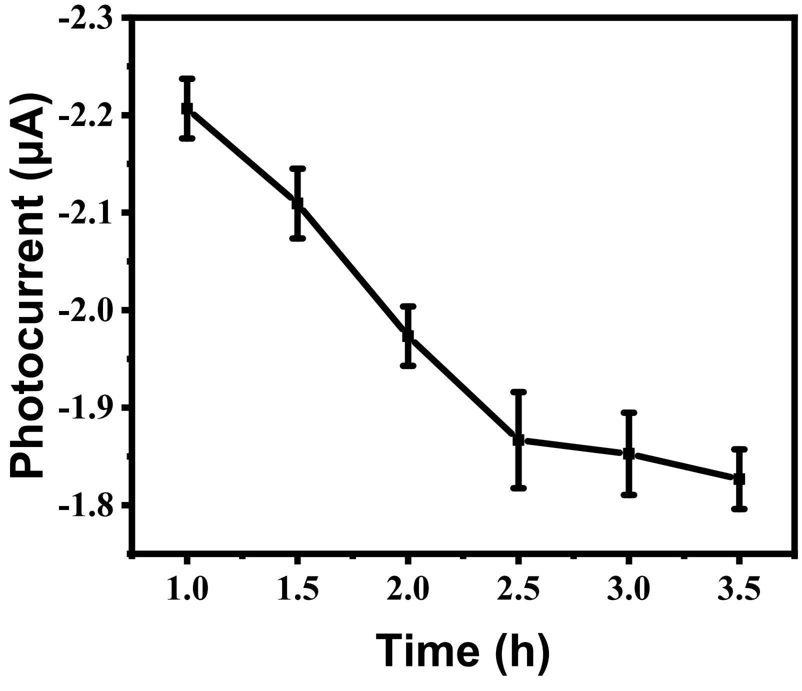

Figure S10. Relationship between photocurrent intensity and the incubation time of PSA aptamer. TTPA-CONs/$Ti_3C_2T_x$ concentration: 3 mg/mL. Aptamer concentration: 5 μM. BSA content: 3 wt%. Incubation time: 50 min. PSA concentration: 10 ng/mL. Incubation time: 120 min. pH of the buffer solution: 7.4. Error bars are derived from the standard deviation of three measurements.

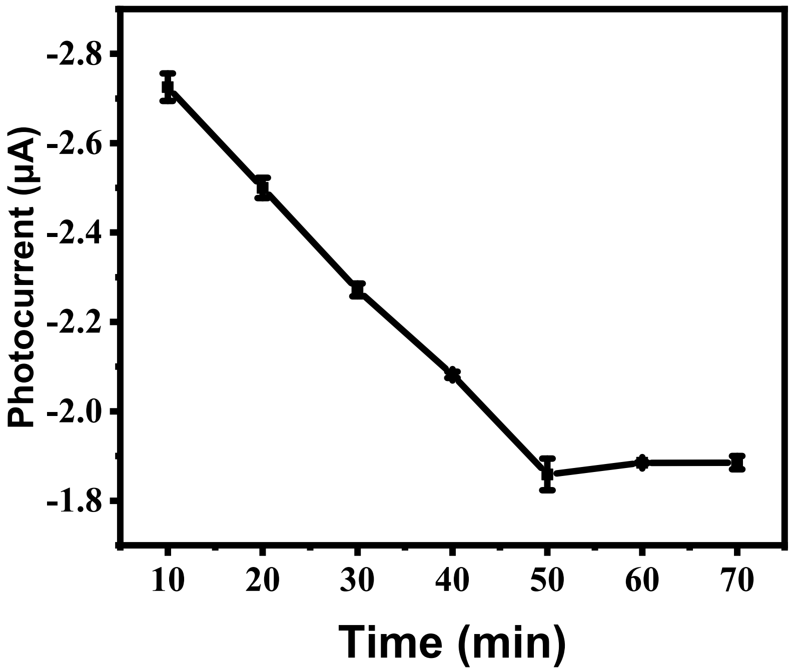

Figure S11. Relationship between photocurrent intensity and the incubation time of BSA. TTPA-CONs/$Ti_3C_2T_x$ concentration: 3 mg/mL. Aptamer concentration: 5 μM. Incubation time: 2.5 h. BSA content: 3 wt%. PSA concentration: 10 ng/mL. Incubation time: 120 min. pH of the buffer solution: 7.4. Error bars are derived from the standard deviation of three measurements.

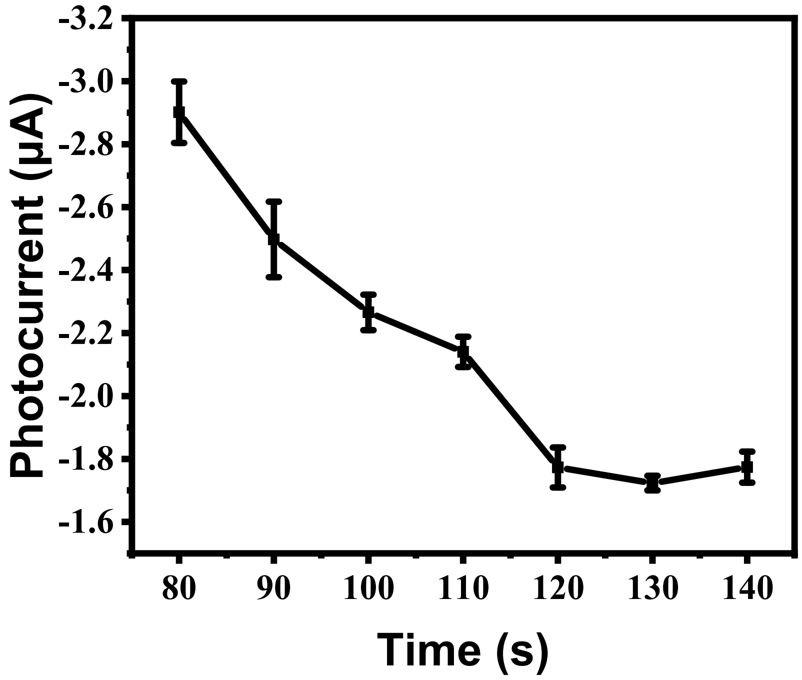

Figure S12. Relationship between photocurrent intensity and the incubation time of PSA. TTPA-CONs/$Ti_3C_2T_x$ concentration: 3 mg/mL. Aptamer concentration: 5 μM. Incubation time: 2.5 h. BSA content: 3 wt%. Incubation time: 50 min. PSA concentration: 10 ng/mL. pH of the buffer solution: 7.4. Error bars are derived from the standard deviation of three measurements.

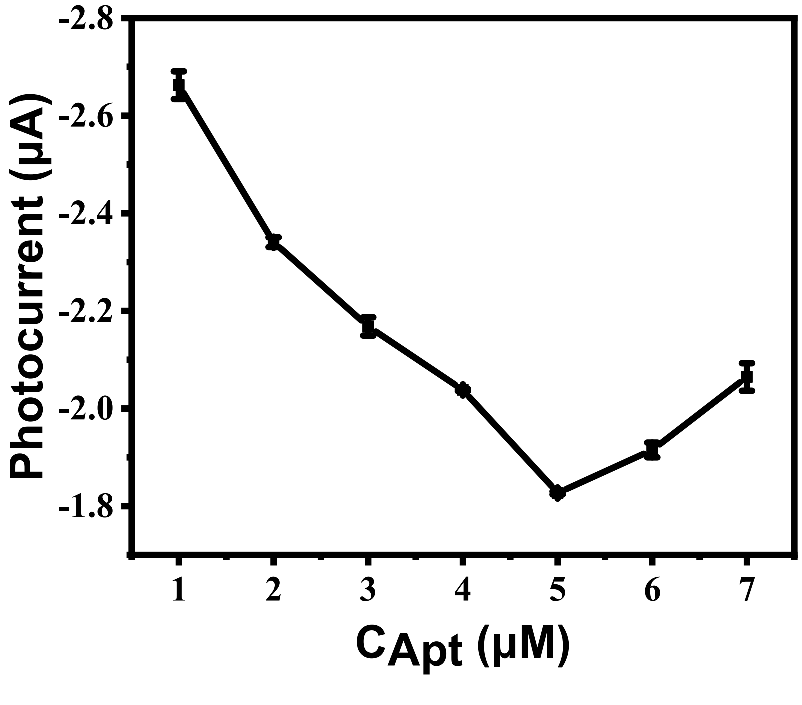

Figure S13. Relationship between photocurrent intensity and the concentration of PSA aptamer. TTPA-CONs/$Ti_3C_2T_x$ concentration: 3 mg/mL. PSA aptamer incubation time: 2.5 h. BSA content: 3 wt%. Incubation time: 50 min. PSA concentration: 10 ng/mL. Incubation time: 120 min. pH of the buffer solution: 7.4. Error bars are derived from the standard deviation of three measurements.

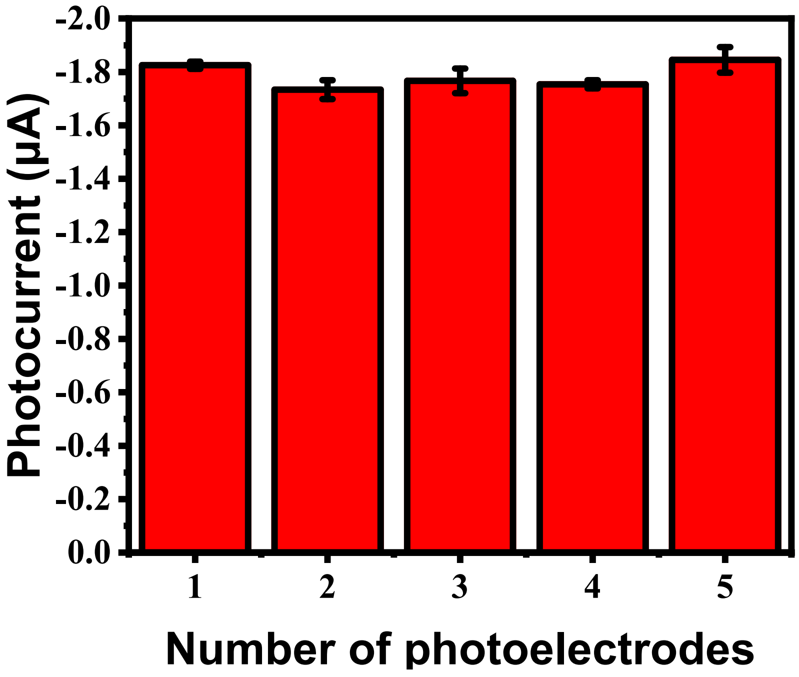

Figure S14. Reproducibility of 5 replicate sensors. TTPA-CONs/$Ti_3C_2T_x$ concentration: 3 mg/mL. Aptamer concentration: 5 μM. PSA aptamer incubation time: 2.5 h. BSA content: 3 wt%. Incubation time: 50 min. PSA concentration: 10 ng/mL. Incubation time: 120 min. pH of the buffer solution: 7.4. Error bars are derived from the standard deviation of three measurements.

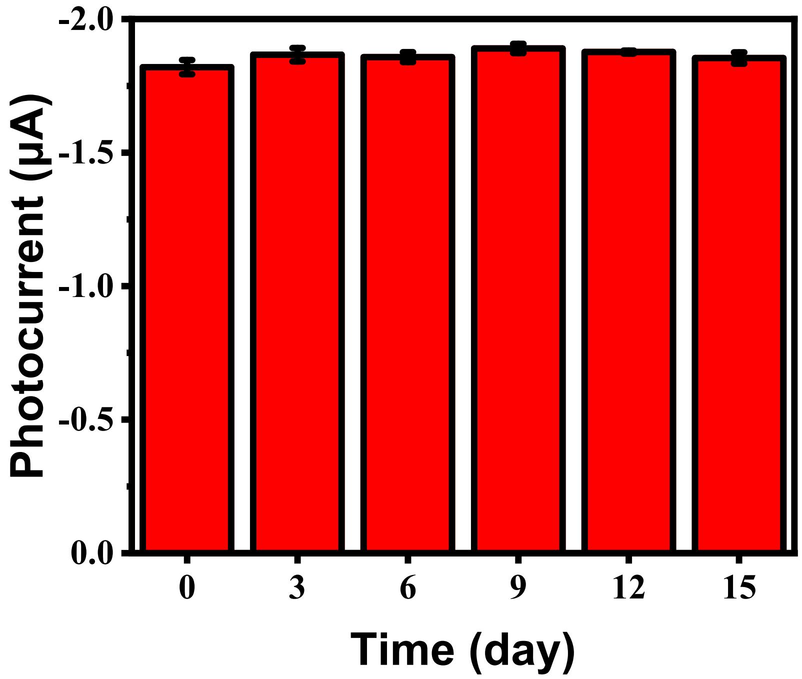

Figure S15. Photocurrent response of PEC sensors with storage time. TPA-CONs/$Ti_3C_2T_x$ concentration: 3 mg/mL. Aptamer concentration: 5 μM. PSA aptamer incubation time: 2.5 h. BSA content: 3 wt%. Incubation time: 50 min. PSA concentration: 10 ng/mL. Incubation time: 120 min. pH of the buffer solution: 7.4. Error bars are derived from the standard deviation of three measurements.

Table S1. Comparison of Different Methods for PSA Determination.

Method	Linear range	Detection limit	Reference
ECL	0.001-100 ng/mL	0.44 pg/mL	[1]
ECL	0.01-10 ng/mL	9.2 pg/mL	[2]
Electrochemical	0.05 -50 ng/mL	28 pg/mL	[3]
Electrochemical	0.001-5 ng/mL	0.31 pg/mL	[4]

Electrochemical	0.0001-50 ng/mL	0.03 pg/mL	[5]
PEC	0.001-10 ng/mL	0.6 pg/mL	[6]
PEC	0.005-20 ng/mL	1.5 pg/mL	[7]
PEC	0.0001-50 ng/mL	6.16 fg/mL	[8]
PEC	0.001-10000 ng/mL	0.3 pg/mL	This work

Table S2. Determination of PSA in bovine serum by the proposed sensor (n = 3).

Added (ng)	Found (ng)	Recovery (%)	RSD (%)
0.1	0.0997	99.7	0.72
1	1.016	101.6	1.76
10	10.32	103.2	0.89
100	94.3	94.3	1.45
1000	1012	101.2	1.35

References

1. Yang, C.; Guo, Q.; Lu, Y.; Zhang, B.; Nie, G. Ultrasensitive "signal-on" electrochemiluminescence immunosensor for prostate-specific antigen detection based on novel nanoprobe and poly (indole-6-carboxylic acid)/flower-like Au nanocomposite. *Sens. Actuators B Chem.* **2020**, *303*, 127246.

2. Zhao, Y.; Zheng, F.; Shi, L.; Liu, H.; Ke, W. Autoluminescence-Free Prostate-Specific Antigen Detection by Persistent Luminous Nanorods and Au@Ag@SiO_2 Nanoparticles. *ACS Appl. Mater. Interfaces* **2019**, *11*, 40669-40676.

3. Zhao, Y., Cui, L.; Sun, Y.; Zheng, F.; Ke, W. Ag/CdO NP-Engineered Magnetic Electrochemical Aptasensor for Prostatic Specific Antigen Detection. *ACS Appl. Mater. Interfaces* **2019**, *11*, 3474-3481.

4. Fan, D.; Li, N.; Ma, H.; Li, Y.; Hu, L.; Du, B.; Wei, Q. Electrochemical immunosensor for detection of prostate specific antigen based on an acid cleavable linker into MSN-based controlled release system. *Biosens. Bioelectron.* **2016**, *85*, 580-586.

5. Dai, L.; Li, Y.; Wang, Y.; Luo, X.; Wei, D.; Feng, R.; Yan, T.; Ren, X.; Du, B.; Wei, Q. A prostate-specific antigen electrochemical immunosensor based on Pd NPs functionalized electroactive Co-MOF signal amplification strategy. *Biosens. Bioelectron.* **2019**, *132*, 97-104.

6. Cao, J.; Dong, Y.; Ma, Y.; Wang, B.; Ma, S.; Liu, Y. A ternary CdS@Au-g-C_3N_4 heterojunction-based photoelectrochemical immunosensor for prostate specific antigen detection using graphene oxide-CuS as tags for signal amplification. *Anal. Chim. Acta.* **2020**, *1106*, 183-190.

7. Zhao, J.; Wang, S.; Zhang, S.; Zhao, P.; Wang, J.; Yan, M.; Ge, S.; Yu, J. Peptide cleavage-mediated photoelectrochemical signal on-off via CuS electronic extinguisher for PSA detection. *Biosens. Bioelectron.* **2020,** 150, 111958.

8. Sun, X.; Li, C.; Zhu, Q.; Chen, J.; Li, J.; Ding, H.; Sang, F.; Kong, L.; Chen, Z.; Wei, Q.; A novel ultrasensitive sandwich-type photoelectrochemical immunoassay for PSA detection based on dual inhibition effect of Au/MWCNTs nanohybrids on N-GQDs/CdS QDs dual sensitized urchin-like TiO_2. *Electrochim. Acta* **2019**, *333*, 135480.
